# The magnitude of sub-optimal child spacing practices and its associated factors among women of childbearing age in Wolaita zone, Sodo Zuria District, Southern Ethiopia: community based cross-sectional study

**DOI:** 10.11604/pamj.2023.44.62.34493

**Published:** 2023-02-01

**Authors:** Biruk Meskele, Amene Abebe Kerbo, Daniel Baza, Mesfin Markos Kacharo

**Affiliations:** 1 Department of Reproductive Health, Wolaita Sodo University, Wolaita Sodo, Ethiopia,; 2 Department of Reproductive Health and Human Nutrition, Wolaita Sodo University, Wolaita Sodo, Ethiopia,; 3 Department of Pediatrics and Neonatal Nursing, Wolaita Sodo University, Wolaita Sodo, Ethiopia,; 4 Department of Midwifery, Wolaita Sodo University, Wolaita Sodo, Ethiopia

**Keywords:** Birth spacing, birth interval, fertility rate, Sodo Zuria District, Southern Ethiopia

## Abstract

**Introduction:**

sub-optimal birth spacing is higher in sub-Saharan countries including Ethiopia. It can affect the economic, political and social aspects of a given country. Therefore, this study aimed to assess magnitude of sub-optimal child spacing practice and associated factors among childbearing women in Southern Ethiopia.

**Methods:**

a community based cross-sectional study was conducted from July to September 2020. A random sampling technique applied to select kebeles, and systematic sampling was employed to recruit study participants. Data were collected by pretested and interviewer administered questionnaire through face-to-face interviews. Data cleaned and checked for completeness, and analyzed by SPSS version 23. A p-value of < 0.05 was considered as cutoff point to declare the strength of statistical association with 95% of CI.

**Results:**

magnitude of sub-optimal child spacing practice was 61.7% (CI: 57.7: 66.2). Not attending formal education (AOR= 2.1 (95% CI: 1.3, 3.3), family planning utilization for less than 3 years (AOR= 4.0 (95% CI: 2.4,6.5), being poor (AOR= 2.0 (95% CI: 1.1, 4.0), breastfeeding of less than 24 months (AOR= 3.4 (95% CI: 1.6,6.0); having more than 6 children (AOR= 3.1 (95% CI: 1.4,6.7); and waiting time ≥30 minutes (AOR= 1.8 (95% CI: 1.2,5.9) were predictors of sub-optimal birth spacing practices.

**Conclusion:**

sub-optimal child spacing was relatively high among the women of Wolaita Sodo Zuria District. Improving utilization of family planning, expanding all inclusive adult education, delivering community based continuous education on optimum breast-feeding practice, involving women in income generating activities, and facilitated maternal services were recommended to fill the identified gap.

## Introduction

Birth spacing is the length of time between two successive life births [1]. Demographers gave attention to inter-birth intervals because of the implications for fertility and maternal and child health [2]. The optimal birth spacing initiative is established under the auspices of USAID to place optimal birth spacing on the global leadership priority agenda to institute an optimal birth spacing recommendation of a minimum of three to five years at the policy, programmatic, and behavioral levels. A longer time between births allows the next pregnancy and birth to be more likely to occur at full gestation or not to be premature. Family planning programs have advocated three or more year intervals between births for child health and infant survival [3].

A high fertility rate is one of the biggest concerns for developing countries throughout the world because it can affect the overall economic, political, and social aspects of a given country. Currently, there is an improvement in the total fertility rate reduction from 5.8 in 2000 to 4.6 in 2016 but is not still achieved the target in 2020 [4,5]. However, still, now Africa has one of the highest rates of fertility and the largest percentage of population growth in the world which imped factor on the progress of the reduction of under-five children [6]. Ethiopia is a country with a total fertility rate of 4.6 (2.3 for urban and 5.2 for rural) and the prevalence of contraceptive use in the country is only 35% which can aggravate sub-optimal birth spacing.

Evidence indicates that there is a direct and indirect relationship between sub-optimal birth spacing and maternal and child morbidity and mortality [7]. Sub-optimal birth spacing is related to neonatal and infant mortality by 3 fold [8], and under-five mortality by twofold [9]. Short birth intervals are also associated with adverse neonatal outcomes such as low birth weight, and being small for gestational age [3,10]. Too short an inter-birth interval can lead to a confrontational maternal condition resulting in the third trimester bleeding, the premature rupture of membranes, anemia, preeclampsia, and puerperal endometritis [11].

Studies indicate that factors like maternal education, maternal age, early marriage, duration of breastfeeding practice, as well as poor knowledge, attitude and practice towards modern contraceptives use and different socio-demographic factors, were factors for the occurrence of sub-optimal birth spacing [12-14]. Shreds of evidence suggest that the practice of sub-optimal birth spacing practice was worse in Ethiopia and it varies from area to area. Understanding the practice and factors that influence women´s birth spacing is essential for the government and stakeholders to tackle the negative consequence of sub-optimal birth spacing practice in the study setting. Therefore, this study is aimed to fill the gap in sub-optimal birth spacing practice and its associated factors among childbearing age women in Wolaita zone, Sodo Zuria District, Southern Ethiopia.

## Methods

**Study setting, design, and period:** this study was conducted in Sodo Zuria District, Wolaita zone, Southern Ethiopia. According to the 2019 Wolaita Zone Sodo Zuria district administrative health office report, Sodo Zuria District has 25 kebeles with a total population of 130,801. The population comprised 65,139(49%) males and 65,662(51%) females of which 30,477 were women in the reproductive age group (15-49 years). According to the report, there were 20,418 under-five children and 4173 infants. The district has 6 health centers, 3 medium, and lower-level clinics, and 10 health posts. A community-based cross-sectional study design was employed in Wolaita Zone, Sodo Zuria District from July to September 2021.

**Population:** women who are in the childbearing age group were the source population. All women living in the study area for greater than 6 months, in the reproductive age group (15-49), who have at least two consecutive live births, and who gave the last birth within the last 3 years were included in this study. All women having a mental illness, not voluntarily participating in the study, and history of any type of abortion between the two successive births were excluded.

**Sample size determination and sampling procedure:** the sample size was determined using a previously conducted study in Ethiopia where the proportion of reproductive-age women who practiced sub-optimal birth spacing in Serbo Town, Oromia Region (P=0.599) [12]. The actual sample size was calculated using single population proportion formula with the assumption of 95% C.I., 80% power, design effect 1.5, 5% margin of error, and a 10% non-response rate, the final sample size became 609.

**The sampling procedure:** eight kebeles were selected from the total of 25 kebeles found in the Sodo Zuria District. The kebeles were selected by using a random sampling technique. The calculated sample size was allocated proportionally to the size of the population in each kebeles. Households with two or more children were selected by marking during census from the selected kebeles. The required number of the study participants for each kebele (the lowest administrative structure in Ethiopia) was calculated by considering the average number of reproductive-aged women found in each Kebeles: Waja Kero Kebele 609*504/4846=63, Waja Shoya Kebele 609*406/4846=51, Waraza Lasho kebele 609*935/4846=118, Dalbo Atwaro kebele 609*424=53, Dalbo Wogene Kebele 609*600/4846=75, Lasho Ketema Kebeles 609*458/4846=58, Dalbo Ketema 609*497/4846 =63, Gurumo Woyde 609*1022/4846=128. Then, the systematic sampling interval (K=4846/609=8) was used to select study participants. The first study respondent was selected by random sampling method.

### Variables of the study

**Dependent variables:** sub-optimal child spacing practices.

### Independent variables

**Socio-demographic and economic variables:** age, maternal educational level, husband education, religion, maternal occupation, husband occupation, age at first marriage, economic status, residence.

**Maternal knowledge:** birth interval, advantages, and disadvantages of the birth interval, the duration of the birth interval, breastfeeding.

**Birth history:** place of delivery, parity, multiple pregnancies, family composition, sex preference, death of preceding child, stillbirth

**Maternal obstetric and reproductive health service utilization:** availability of contraceptives, distance to the health facility, waiting time for maternal services, contraceptive utilization, duration of contraceptive utilization, fear of family planning side effects, breastfeeding (exclusive and duration of breastfeeding), postpartum amenorrhea, postpartum abstinence, postnatal care utilization, postpartum abstinence, postpartum amenorrhea.

### Operational definitions

**Birth interval:** is the time between the current live birth and the preceding live birth [15].

**Long birth interval:** birth interval greater than 60 months [7].

**Optimal birth interval:** refers to 36-60 months between births [7].

**Sub-optimal birth spacing:** birth interval less than 36 months after the preceding live birth [7].

**Data collection tools and procedures:** data collection tools were prepared after an extensive review of published literature on a similar topic. Then, the tools were first prepared in English, translated to the local language (Wolaitato), and translated back to the English language. The tools consisted of socio-demographic and economic characteristics of the women, knowledge, and practice of birth spacing, utilization of contraception methods and other reproductive health services, breastfeeding practices, and obstetric and birth history of the women.

**Data quality assurance:** eight diploma-level nurses who know the local language (Wolaitato) were recruited for data collection. Two BSc professional nurses and two midwives were recruited for supervision. Supportive supervision was carried out by the principal investigator to check the completeness and consistency of the data collected. The questionnaire was prepared in the English version and translated into the local language (Wolaitato) and then back-translated into English to maintain its consistency. Two days of training were given on the process of data collection for data collectors. A pretest was done by Shola Borkoshe kebele on 5% of the study population before starting data collection. The clarity of questions and validity of the instrument was done after the pretest.

**Data processing and analysis procedure:** the data were visually checked for completeness, coded, and entered into Epinfo version 7.2 software and then exported to SPSS version 23.0 for analysis. Descriptive statistics were used to give a clear picture of the dependent and independent variables. Frequency distributions of the variables were worked out using tables and figures. Bivariable logistic regression analysis was used to see the significance of the association between the dependent and each independent variable. Candidate variables for the multivariate binary logistic regressions were identified at p-value < 0.20. Variables that had a significant association with the outcome variable during bi-variable logistic regression analysis were introduced into multivariable binary logistic regression. Predictors of sub-optimal child spacing practices were declared at p-value < 0.05 with 95% CI.

**Ethics approval and consent to participate:** ethical clearance letter and approval were obtained from Wolaita Sodo University Institutional Review Board. A letter of cooperation from Wolaita Sodo University College of Health Science and Medicine was written to Wolaita Sodo Zuria District health office. Then, Wolaita Sodo Zuria District health office communicated with respective kebeles. Written consent was obtained from study participants after explaining the study objectives and procedures, and their right to refuse not to participate in the study at any time was assured. Confidentiality of the information was ensured by coding. The interview was undertaken privately in an area separated from others. Only authorized individuals were given access to the raw data collected from the field.

## Results

**Socio-demographic characteristics of childbearing age women´s in Wolaita Sodo Zuria District, Southern Ethiopia 2021 (n=609):** the response rate of this study was 98.6%. The mean age of the respondents was 28.27 (SD=5.15). Five hundred sixty (93.2%), 412(68.6%), and 396 (65.9%) were married, protestant Christianity followers, and rural dwellers respectively. One-fifth of the study participants were aged under 18. One hundred eighty (30%) did not attend any formal education while only 20 (3.3%) of the husbands had not attended formal education. Most of the study participants were Wolaita in ethnicity 524(87.2%). One-tenth of the study participants were categorized under the poorest wealth indexes (Table 1).

**Table 1:** socio-demographic characteristics of childbearing age women’s in Sodo Zuria District, Wolaita Zone, Southern Ethiopia

Variables		Frequency	Percentage
Marital status	Married	560	93.2%
	Other	41	6.8%
Age at first marriage	<18	121	20.1%
	≥18	480	79.9%
Religion	Protestant	412	68.6%
	Orthodox	158	26.3%
	Other	31	5.2%
Ethnicity	Wolaita	524	87.2%
	Gamo Gofa	40	6.7%
	Other	37	6.2%
Maternal education	No formal education	180	30%
	Formal education	421	70%
Maternal occupation	Housewife	450	74.9%
	Employee	78	13%
	Merchant	73	12.1%
Wealth Index	Poorest	121	20.1%
	Poor	119	19.8%
	Middle	125	20.8%
	Rich	119	19.8%
	Richest	117	19.5%

**Reproductive health service utilization, birth history, and breastfeeding practices of the study participants in Wolaita Sodo Zuria District, Southern Ethiopia (n=609):** regarding the reproductive characteristics of the study participants, the majority had received information on optimal birth spacing 494 (82.2%), exclusively breastfed 438 (72.9%), and breastfed her child less than 24 months 491 (81.7%). The majority 89.2 % have used any family planning methods for less than three years (49.3%), and 344 (61.8%) of women used injectables (Table 2).

**Table 2:** reproductive health service utilization, birth history and breastfeeding practices of the study participants in Sodo Zuria District, Southern Ethiopia (n=609)

Variables		Frequency	Percentage
Duration of birth spacing	<3years	260	43.3%
	3-5 years	275	45.8%
	≥6 years	66	11%
Family planning methods used	Pills	43	5.6%
	Injectables	344	61.8%
	Implants	112	18.5%
	Intrauterine contraceptive device	63	9.3%
	Other	39	4.8%
Duration of family planning use	< 3 years	286	49.3%
	3-5 years	149	23.6%
	≥6 years	166	27.1%
Exclusive breastfeeding for preceding baby	Yes	438	72.9%
	No	163	17.1%
Duration of breastfeeding for preceding baby	0-23 months	491	81.7%
	≥24 months	110	18.3%
Exclusive breastfeeding for preceding baby	≤5 months	44	7.8%
	6 months	552	91.8%
	7 months	5	0.8%
Number of alive children	<2 children	245	40.8%
	children	279	46.4%
	≤5 children	77	12.8%

**Prevalence of child spacing practices among women of reproductive age in Wolaita Sodo Zuria District, Southern Ethiopia:** this study revealed that 371(61.7%) with (95%CI: 57.7: 66.2) of the participants practiced sub-optimal child spacing and 233(38.3%) of them were not practicing the optimal child spacing practices.

**Figure 1:**
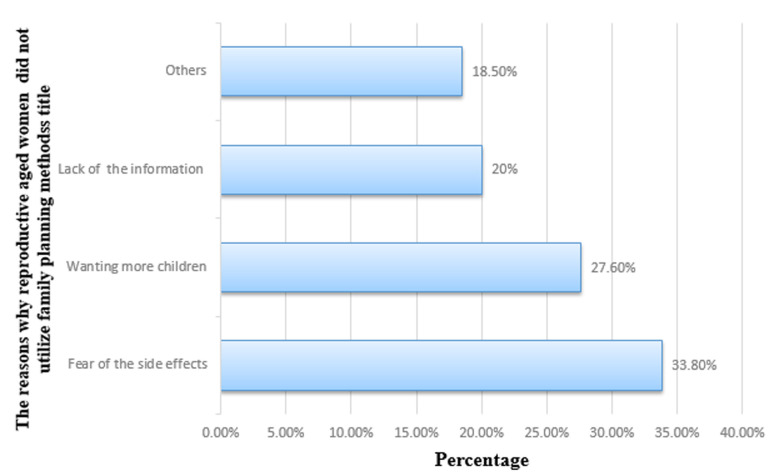
the reasons why reproductive-aged women in Wolaita Sodo Zuria District did not utilize family planning methods, Southern Ethiopia

**The reasons why reproductive-aged women in Wolaita Sodo Zuria District did not utilize family planning methods, Southern Ethiopia:** approximately 22 (33.8 %) of women did not utilize family planning due to the fear of side effects and 18 (27.6 %) of them were not using the methods because of wanting more children (Figure 1).

**Factors associated with sub-optimal child spacing practice among women of childbearing age in Wolaita Sodo Zuria District, Southern Ethiopia:** in order to identify the possible factors for sub-optimal child spacing practices, Bivariable logistic regression analysis was done. Based on the present study, marital status ( COR =1.7, 95 % CI=0.9-3.3), age at first marriage (COR=0.6, 95% CI= 0.4-0.9), residence (COR=1.5, 95% CI=1.1-2.1), maternal education (COR=1.6, 95%=1.1-2.3), maternal occupation ( COR=1.6, 95 % CI= 1.1-2.3), wealth index (COR=1.6, CI 95 % CI= 1.1-2.3), duration of family planning utilization (COR=3.5, 95 % CI= 2.3,5.5), duration of breastfeeding ( COR= 2.74, 95 % CI=1.78,4.2), availability of contraception (COR= 1.5, 95 % CI= 0.9,2.6), place of delivery for preceding baby (COR= 1.3, 95 % CI= 0.8-2.0), sex preference of the previous child (COR= 1.6, 95 % CI= 1.1,2.3), sex of the previous child (COR= 1.5, 95 % CI= 1.1,2.1), waiting time (COR=1.7, 95 % CI= 1.22-2.42), and parity (COR=2.8 95% CI= 1.5-5.2) were factors associated with sub-optimal child spacing practices. Duration of family planning for less than 3 years (AOR: 4.0, 95% CI: 2.4,6.5), mothers who had no formal education (AOR; 2.1 (95% CI: 1.3, 3.3), mothers with low monthly income (AOR: 2.0 (95% CI: 1.1, 4.0), duration of breastfeeding less than 24 months (AOR: 3.4, 95% CI: 1.6,6.0), number of alive children greater than 6 (AOR= 3.1, 95 % CI=1.4,6.7) and waiting time for maternal service greater than 30 minutes (AOR: 1.8, 95% CI: 1.2,5.9) were identified as the significant and independent factors of sub-optimal child spacing practices in multiple logistic regression analysis (Table 3).

**Table 3:** factors associated with sub-optimal child spacing practice among childbearing age women in Sodo Zuria District, Southern Ethiopia

Variables		Short birth spacing		COR (95% CI)	P-value	AOR(95% CI)
		No	Yes			
Maternal education	No formal education	55	125	1.6(1.1-2.3)	0.011	2.1(1.3,3.3)
	Formal education	175	246	1		1
	Others	145	98	0.8(0.52-1.23)	0.31	
Wealth index	Poorest	40	81	1.5(0.9,2.5)	0.125	2.0(1.1,4.0)
	Poor	52	67	0.9(0.6,1.6)	0.881	1.2(0.6,2.2)
	Middle	48	77	1.2(0.7,2.0)	0.493	1.5(0.8,2.9)
	Rich	40	79	1.5(0.8,2.5)	0.150	1.9(0.9,3.2)
	richest	50	67	1		1
How long used family planning (N=536)						
	<3 years	65	199	3.5(2.3,5.5)	0.000	4.0(2.4,6.5)
	3-5 years	58	69	1.4(0.9,2.2)	0.182	1.2(0.7,2.0)
	≥6	78	67	1		1
Duration of breast feeding for preceding baby	0-23 months	166	325	2.74(1.78,4.2)	0.031	3.4(1.6,6.0)
	≥24 months	64	46	1		1
Number of alive birth	≤	99	145	1		1
	3-5 children	116	163	0.9(0.67-1.35)	0.80	0.9(0.6,1.4)
	≥6 children	15	62	2.8(1.5-5.2)	0.001	3.1(1.4,6.7)
Waiting time for services	<30 minutes	153	199	1		1
	≥30 minutes	77	172	1.7 (1.22-2.42)	0.002	1.8(1.2,5.9)

## Discussion

The prevalence of sub-optimal child spacing was in agreement with the previous studies in Nigeria (65.9%) [13] and Jimma (59.9%) [12]; however, the prevalence was higher than in studies done in Bangladesh (24.6) [16], Pakistan (22.9) [17], Iran (28.5) [18], Tanzania (48.4) [19], and northern Ethiopia (23.3) [20]. The higher prevalence might be due to variations in socio-economic and socio-demographic characteristics, and accessibility of health services. Moreover, the higher prevalence of sub-optimal child spacing in this study when compared to northern Ethiopia might be due to the lower prevalence of modern contraceptive utilization [4].

Women´s education status was associated with sub-optimal child spacing. The odds of sub-optimal child spacing were higher for uneducated women compared to educated ones. This finding was in line with studies done in Iran [21], Gedeo zone southern Ethiopia [22], Arba Minch southern Ethiopia [23], and Illubabor central Ethiopia [24]. This might be because educated women were more likely to use family planning. Moreover, they might have a higher level of healthcare-seeking behaviors when compared to uneducated ones [25]. This finding might imply the need of empowering women by increasing educational opportunities and expanding the availability of basic education at the community level. Socio-economic characteristic was one of the factors that affected sub-optimal child spacing in this study. This finding was comparable to studies in Bangladesh [16,26], in the multilevel analysis of Ethiopia EDHS [27], Gedeo zone southern Ethiopia [22], and southern Ethiopia [23]. This is plausible because women from the poorest economic class have a greater chance of practicing sub-optimal child spacing because of a lack of easy access to maternal services because of the inability to pay for services and transportation [23]. Moreover in this study, the majority (75%) of women in the poorest economic class were from remote areas.

Several studies identified contraception utilization as one of the powerful predictors of the child spacing practice; these included. Studies in Pakistan [17], South Iran [28], Northern Ethiopia [20], Dodota Woreda, Arsi Zone, and Arba Minch [23,29]. However, in this study duration of modern contraceptive utilization was identified as a predictor for child spacing. The odds of practicing sub-optimal child spacing were higher for women who utilized contraception for less than three years compared to their counterparts. This finding is in agreement with the WHO recommendation on the promotion of long-acting family planning for women to increase the level of optimal child spacing [30]. However, national strategies of family planning also recommend the utilization of contraception [31], and the FMOH (2016-2020) reproductive strategies also target an increase in contraceptive acceptance rate from 35 to 42% [5]. Moreover, this finding implies the need for government and stakeholder attention to promoting adequate duration of the utilization of contraception, rather than simply promoting the utilization of contraception.

Duration of breastfeeding was one of the identified determinants for sub-optimal child spacing in this study. The odds of practicing sub-optimal child spacing were lower for women who had breastfed their previous child less than 24 months when compared to those women who breastfed their previous child at least 24 or above months. This finding was supported by studies done in Nigeria, Northern Ethiopia, Arba Minch, and Arsi [20]. This is obviously due to optimal breastfeeding functioning as a contraceptive method to prevent pregnancy. Moreover, the reason for this finding could be children who breastfeed for a long duration could be well and healthy [32]. This might decrease the need for the next child, which in turn reduces sub-optimal child spacing. However, the optimal duration of breastfeeding must be complemented with contraceptive utilization to decrease sub-optimal child spacing.

The number of live children also affects the level of sub-optimal child spacing. Women who had a higher number of live children were more likely to have practiced sub-optimal child spacing in this study. This finding was in contrast to a study done in Bangladesh where those who achieve higher-order parities were less likely to experience short birth intervals [16]. This discrepancy might be due to variations in the source of data, sample size, time variation, and study design. The former one used secondary data, a large sample size, and a retrospective cross-sectional study design. Waiting time for accessing maternal health services was one of the predictors for sub-optimal child spacing in this study. Women who have a waiting time of more than 30 minutes for health services were more likely to have practiced sub-optimal birth spacing when compared to those women who had waited less than 30 minutes. The reason for this finding might be individuals who spent a longer waiting time for accessing health services may not attend their next visit to health services [33-3,5] because a longer waiting time reduces client satisfaction and subsequent service utilization [36,37]. This in turn reduces the utilization of maternal services that reduce sub-optimal child spacing. This finding indicated that the need for improvement in the implementation of compassionate, respectful care for maternal health services at health facilities because the timely provision of service is attending to client needs and is one of the components of compassionate respectful; and caring [38]. Moreover, the government should give attention to reducing delays at the health facilities level.

## Conclusion

The prevalence of short child spacing practice was high (61.7%) when compared to several studies. Not attending formal education, family planning utilization of fewer than 3 years, being poor, breastfeeding of fewer than 24 months, having more than 6 children, and waiting time for maternal service of ≥30 minutes were independent predictors of short child spacing practices among women´s of childbearing age in the study setting. Improving the utilization of modern family planning, providing continuous education on optimum breastfeeding practice, creating opportunities for mothers to be involved in income-generating activities, and facilitating maternal services in health facilities may help to decrease sub-optimal child spacing practices.

**Strength and limitations:** since this study was community-based, thus the findings of the study could be generalized to the target population. Finally, there could be a recall bias since women were asked for information about events that occurred in the distant past through different life events were used to memorize the past. We used a large sample size in the current study which increases the generalizability of the finding to the study setting.

### What is known about this topic

- *Encouraging the duration of birth spacing for a minimum of two years results in the significant reduction of under-five mortality in Ethiopia;*
- *Birth spacing is recognized as an essential intervention for mothers, newborns and infants;*
- *World Health Organization suggests at least 24 months after a live birth-to-pregnancy and 33 months’ birth to birth intervals to reduce unwanted maternal, perinatal, neonatal and infant outcomes*.

### What this study adds

- *The prevalence of optimal birth interval in this study is significantly lower than in other similar studies;*
- *Not attending formal education, family planning utilization for less than 3 years, being poor, breastfeeding of less than 24 months, having more than 6 children and waiting time for maternal service for ≥30 minutes were predictors of birth spacing practices in the study setting;*
- *Improving utilization of modern family planning, providing continuous education on optimum breastfeeding practice, creating opportunities for mothers to be involved in income-generating activities, and facilitated maternal services in health facilities may help to decrease sub-optimal child spacing practices*.
